# Plastic Deformation of High Density Polyethylene with Extended-Chain Crystal Morphology

**DOI:** 10.3390/polym15010066

**Published:** 2022-12-24

**Authors:** Alina Vozniak, Zbigniew Bartczak

**Affiliations:** Centre of Molecular and Macromolecular Studies, Polish Academy of Sciences, Sienkiewicza 112, 90-363 Łódź, Poland

**Keywords:** semicrystalline polymer, deformation mechanisms, deformation instabilities, microbuckling, kinks, polyethylene, extended-chain crystals

## Abstract

Samples of polyethylene with extended-chain crystal morphology, obtained by crystallization under high pressure, were subjected to uniaxial compression to various strains. Accompanying structural changes were analyzed using scanning electron microscopy. At the true strain of e = 0.2–0.3 the microbuckling instability was observed in longitudinally loaded lamellae, resulting in the formation of angular kinks. This induced a rapid reorientation of the lamellae, facilitating their further deformation by crystallographic slip. Microbuckling instability was found to occur earlier than in samples with folded-chain crystal morphology (e = 0.3–0.4) due to a smaller ratio of the amorphous to crystalline layer thickness. SEM observations demonstrated that the microbuckling instability begins with small undulation in long lamellae. Sharp angular lamellar kinks develop from the initial undulation through intense plastic deformation by crystallographic slip along the chain direction. The same slip system was found to operate throughout the kink, including the tip region as well as both limbs. In contrast to thin folded-chain lamellae that often undergo fragmentation during deformation, the thick extended-chain lamellae deform stably by chain slip and retain their continuity up to high strains, e > 1.6. This stability of deformation is related to the large thickness of extended-chain lamellae.

## 1. Introduction

Semicrystalline polymers show an exceptional ability to large-scale plastic deformation. This is considered one of their most important features. Therefore, the plastic deformation behavior of semicrystalline polymers has been extensively studied; see reviews—e.g., [1,2,3,4,5]. Nevertheless, there still remain some unexplored areas in this field. This is because the deformation of semicrystalline polymers has turned out a complex sequential process involving all elements of their complicated, multi-level morphology and involving a wide variety of mechanisms that can be triggered or terminated at different stages. The exceptional deformability of semicrystalline polymers is mainly attributed to their distinctive morphology, at the basic level consisting of alternating thin amorphous and crystalline layers that are coupled at interfaces by covalent bonds owing to numerous chains crossing the crystalline-amorphous interface. Such molecular links ensure very strong bonding of adjacent crystalline (hard) and amorphous (soft) layers. Moreover, these chains, passing through amorphous layer may connect neighboring lamellae, either directly (tie-molecules) or by entanglements with other chains in the amorphous layer between lamellae. Such molecular links facilitate the load transfer between neighboring lamellae in the stack and therefore, are called stress transmitters (ST) [3]. 

The deformation of crystalline lamellae proceeds primarily by means of crystallographic mechanisms, including the crystallographic slip, which appeared the principal mechanism [1,2,3,4,5]. The deformation of crystalline layers is accompanied and supported by mechanisms active in amorphous layers, mainly the interlamellar shear (also referred to as interlamellar slip). Other reported mechanism are lamellar separation and the rotation of lamellar stacks. The strong interconnection of adjacent crystalline and amorphous layers through ST chains forces their cooperative deformation—obviously, both layers must deform simultaneously and jointly to maintain the integrity of the interface. Consequently, the deformation mechanisms operating at a given stage, in the crystalline and amorphous phases, respectively, have to be fine-tuned due to the mutual feedback between the adjoining layers forced to deform jointly. In some cases, the active mechanism may even be replaced by an alternative mechanism that appears to be more effective at this point. Recent studies demonstrated a very significant role of the amorphous phase and its topology-dependent properties in the process of plastic deformation [6,7,8,9,10,11,12,13]. Amorphous layers were found much more important than merely a compliant medium transmitting the load and adapting to the deformation of crystalline lamellae. They turned out an important and active part of the structure, capable of tuning the deformation of the crystalline phase and, under certain conditions, even controlling the entire deformation process. 

Interactions between amorphous and crystalline components may also induce some instabilities in the deformation process, which, in turn, may result in the opening of new, more effective, but previously inaccessible paths of deformation, and consequently, a profound modification of the entire deformation route. Therefore, the deformation instabilities often appear to be a very important part of the deformation process, facilitating easier strain accommodation. The major deformation instabilities observed in semicrystalline polymers are associated with cavitation (see, e.g., the review [14]), the microbuckling of lamellae [15,16,17,18,19,20,21,22], and their fragmentation due to slip localization [6,20,21,23,24]. Such instabilities often lead to profound structural and morphological changes, such as conversion of the initial lamellar/spherulitic morphology into microfibrillar morphology, usually observed in tension. The above-mentioned instabilities of deformation are largely related to the presence and properties of the amorphous phase layers, their response to stress, as well as interactions with adjacent crystalline lamellae [12], including stress concentrations generated at interface due to ST chains. 

While the cavitation, commonly seen in drawing, has been extensively studied [14], much less attention has been paid to other instabilities such as microbuckling or lamellae fragmentation. Microbuckling is the instability of deformation that results in undulation of lamellae and adjacent interlamellar amorphous layers, which quickly leads to formation of joint folds or angular kinks of lamellae. Microbuckling in semicrystalline polymers is generally analogous to buckling in other layered systems. Folded structures due to buckling of layers have been observed very often in various materials with layered morphology. Buckling was recognized as a common mechanism of deformation, occurring in a variety of layered materials with very different length scales, ranging from kilometers, as in formations of natural rock, to the molecular length scale (nano- and microbuckling), e.g., in smectic liquid crystals [12]. Buckling instability occurs primarily in response to the compressive load parallel to layering, but in some materials, such as liquid crystals, copolymers or semicrystalline polymers (exhibiting strong interlayer coupling and high Poisson’s ratio), it has also been observed under a tensile force acting perpendicular to the layers [15,17,18,19]. Microbuckling in semicrystalline polymers occurs in those lamellar stacks that have been initially oriented parallel to the principal compression direction (i.e., the load direction is parallel to layering during compression or perpendicular to it during tension). The lamellae of such specific orientation are not able to deform according to the usual, relatively easy crystallographic mechanisms because the corresponding resolved shear stresses in the potential slip planes are too low, well below critical stresses required for activation of any of these mechanisms. Consequently, the deformation is locally locked in the immediate vicinity of such lamellae and the stresses increase with advance of strain. In such a situation, the stacked lamella can respond to increasing compressive in-plane stress with bending and/or undulation, a phenomenon referred to as microbuckling instability [18,20]. Due to phase connectivity the neighboring lamellae are forced to bend cooperatively, which soon leads to the development of bigger joint folds or angular kinks from the initial small ripple, and may eventually lead to a chevron-like morphology. This mechanism is limited to the specifically oriented lamellae and therefore can accommodate only a minor strain in macroscopically non-oriented material. Nevertheless, it appears greatly important for further deformation, because it causes a rapid and fairly large rotation of the lamellae which were involved in the kink. This increases the resolved shear stress in the potential slip planes in these just reoriented lamellae, which in turn enables the activation of conventional mechanisms such as crystallographic slip there. It opens a new path of relatively easy deformation, which allows to unlock a significant part of the material volume to be deformed through mechanisms similar to those that were previously available only to other lamellae, as e.g., the diagonal lamellae, already oriented favorably for a slip. Krumova et al. [17] claimed that the microbuckling instability is a micromechanism as important as other, widely accepted mechanisms in polymer plasticity since it can activate and/or supplement the other mechanisms as needed. It was found that the transition initiated by microbuckling frequently manifest macroscopically as a low and broad hump in the stress-strain curve, commonly referred to as the second macroscopic yield point [20,21,22,25]. 

The aim of this work was to gain more knowledge and thus deepen the understanding of microbuckling instability and other deformation instabilities that occur during plastic deformation of semicrystalline polymers to high strains, including obtaining direct microscopic evidence of these phenomena and identifying the active mechanisms. Samples of linear polyethylene, crystallized at high pressure resulting in very thick crystalline lamellae, were chosen for this study. It is well known that the crystallization of PE under high pressure produces samples with crystals much thicker than in conventionally crystallized PE [26]. In the phase diagram of PE, there is a certain range of pressure and temperature (above 330 MPa and 215 °C, respectively), in which crystals of pseudo-hexagonal phase are formed from the melt instead of ordinary orthorhombic phase obtained at low pressure [27]. The high mobility of the hexagonal phase allows for substantial thickening of the growing lamellar crystals and the formation of the very thick, so-called extended-chain lamellar crystals [28], the thickness of which may even exceed 1 micrometer, i.e., greater than the average contour length of the chains. A considerable fraction of pre-existing chain entanglements is resolved, which leads to a significant increase of crystallinity [8,29]. The crystals of pseudo-hexagonal phase are transformed to common orthorhombic modification upon cooling without reduction in size. The large dimensions of the lamellae obtained in this way allow for detailed observation of their deformation behavior and identification of the active deformation mechanisms with a relatively simple SEM technique. 

## 2. Materials and Methods

### 2.1. Material and Sample Preparation

Three grades of high density polyethylene (HDPE), provided by BASF (Ludwigshafen, Germany) and Basell (Rotterdam, The Netherlands), were selected for this study. Their molecular characteristic is given in Table 1. Since the polyethylene denoted hereafter as H3 was an experimental grade, supplied as a powder without additives, a mixture of stabilizers and antioxidants was added to it in order to prevent excessive oxidation during processing and crystallization [16,29]. The grades labeled H4 and H5 were commercial ones that already contained the antioxidants and stabilizers added by manufacturer, thus no additional stabilization was needed. 

**Figure 1:**
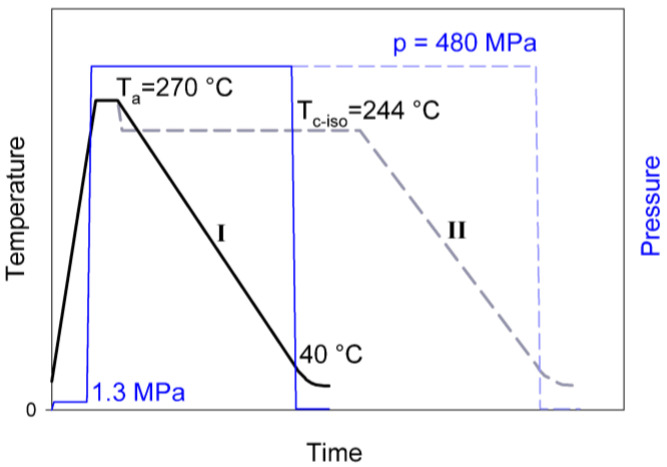
Pressure-temperature protocol of the high pressure crystallization: route I—nonisothermal crystallization (solid lines), route II—isothermal crystallization (dashed lines).

The samples for further deformation studies were obtained by crystallization of the molten polyethylene at an elevated pressure in order to obtain crystals with extended-chain morphology. First, the cylindrical specimens (9.5 mm in diameter and 25 mm long) were formed from H3, H4 or H5 PE by injection molding. The injection rate was very low in order to avoid any orientation. Next, these cylindrical billets were melted and subsequently crystallized at high pressure in a custom made pressure cell. The cell consisted of a barrel of 9.5 mm inner diameter, made of ultrahigh strength steel, and two matching tungsten carbide pistons. The 1 mm thick copper seals placed between sample and the pistons prevented any leakage of the polymer out of the cell. The pressure was applied to the cell by compression in a computer-controlled 100 kN loading frame of the tensile testing machine (Model 5582, Instron, Norwood, MA, USA). The hydrostatic pressure and temperature in the cell were controlled, with an accuracy of 0.2 MPa and 0.5 °C, respectively. The construction of the pressure cell and the details of the procedure of sample crystallization were described elsewhere [16,29]. The pressure-temperature protocol of high-pressure crystallization used in this study is schematically shown in Figure 1. First, the specimen was heated to the temperature T = 180 °C under a mild pressure of 1.3 MPa, which was applied to hermetically seal the cell and ensure good thermal contacts inside the cell. Once the polymer was molten the pressure of 480 MPa was applied and temperature was increased further to T = 260–270 °C. The molten specimen was held at this temperature for 3–5 min to erase its thermal history and residues of orientation. Next, the cell was cooled slowly down to 40 °C at the rate approx. 4 °C/min, still under high pressure (route I). Finally, when temperature decreased below 40 °C, the pressure was released. Additionally, a few specimens of H4 polyethylene were crystallized isothermally by holding the sample at T = 244 °C and p = 480 MPa for 1h prior to cooling to room temperature and pressure release (route II). 

### 2.2. Mechanical Testing

Samples of HDPE with extended-chain crystals (CEPE), obtained by crystallization at high pressure, were subjected to plastic deformation by uniaxial compression. The CEPE samples for the deformation tests were machined out from the high-pressure crystallized HDPE billets in the form of 4 mm thick cylindrical slices (diameter 9.5 mm) with smooth faces. The uniaxial compression was carried using a tensile testing machine (Model 5582, Instron, Norwood, MA, USA) equipped with compression fixture and dynamic extensometer. The smooth specimen faces contacting the polished compression plates of the testing machine were additionally lubricated to minimize friction during the test. All samples were compressed with the constant true strain rate of 0.001 s^−1^ (6 %/min) at room temperature to the desired true strain, ranging from e = 0.2 to 1.6. 

Experimentally determined load—displacement data were recalculated to obtain the true stress—true strain curves. The true stress was calculated from the measured load using the equation:

(1) $$\sigma =\sigma_{n}\lambda^{-2\nu }=\frac{F}{A_{o}}\lambda^{-2\nu }$$

where *σ_n_ = F/A_o_* is the nominal stress (*F*—load, *A_o_*—initial cross-section area of the sample), *λ = CR= h_o_/h* is the compression ratio (*h_o_* and *h = h_o_−*Δ*h* are the initial and actual height of the specimen, respectively), and *ν* is the Poisson’s ratio. The value of *ν* = 0.4 found for HDPE [30] was taken for calculations of the true stress. This value was verified by comparing the dimensions of the deformed specimens with the initial ones. The true strain (Hencky strain) was calculated from the extensometer data with the following formula:

(2) $$e=\ln\left(\lambda \right)=\ln(\frac{h_{o}}{h})=\ln\left(\frac{h_{o}}{h_{o}-\Delta h}\right)$$

where *λ = h_o_/h* is the compression ratio. 

The reference samples of the same shape and prepared from the same materials by slow injection molding, exhibiting conventional folded-chain crystal morphology, were also tested at the same conditions. 

### 2.3. Characterization

#### 2.3.1. Differential Scanning Calorimetry (DSC) 

Thermal analysis the samples was carried out using an indium-calibrated TA 2920 DSC calorimeter (TA Instruments, New Castle, DE, USA). The samples were sealed in standard aluminum sample pans and heated from 25 °C to 200 °C at a constant heating rate of 10 °C/min under dry nitrogen purge of 50 mL/min. The heat of melting Δ*h_m_* was determined by integration of endotherm peak area in the temperature range of 80–160 °C. The degree of crystallinity was calculated using the equation:

(3) $$X_{c}=\frac{\Delta h_{m}}{\Delta h_{f}^{o}}\cdot 100\%$$

where Δ*h_m_* is the heat of melting of measured sample and Δ*h°_f_* = 293 J/g is the heat of fusion per unit volume of 100% crystalline polyethylene [31]. The accuracy of the DSC measurements was approx. 1%. 

For the estimation of the thickness of the crystalline lamellae, the length of the crystalline stem, l*, was calculated from the melting peak temperature, *T_m_*. The Gibbs-Thomson equation was used [32]:

(4) $$l^{*}=\frac{2\sigma_{e}T_{m}^{o}}{\Delta h_{f}^{o}\left(T_{m}^{o}-T_{m}\right)}$$

where *σ_e_* = 9·10^−6^ J/cm^2^ is the lamellar basal surface free energy of PE crystal [33] and *T_m_^o^* = 145.5 °C is the extrapolated equilibrium melting temperature of infinite PE crystal [33]. Because of chain tilt the thickness of the lamellar crystal, *l_c_*, is expected to be smaller than the crystalline stem length l*.

#### 2.3.2. Scanning Electron Microscopy (SEM)

The specimens for microscopic observations were prepared from non-deformed and compressed CEPE-samples by permanganic etching, according to the procedure developed originally by Olley et al. [34]. Prior to etching, the bulk morphology of the sample was exposed by cutting the sample along the plane of interest (usually the plane parallel to the loading direction), with an ultramicrotome (Tesla, Brno, Czechia) equipped with a freshly prepared glass knife. The exposed surfaces were etched at room temperature in the fresh solution composed of 1 wt.% of KMnO_4_, dissolved in a 1:1 vol./vol. mixture of concentrated sulfuric and phosphoric acid. The time of etching was typically 60 min, based on a series of preliminary experiments. The etched samples, carefully washed and then coated with a fine gold layer (Edward Sputter Coater, Edward, Crawley, UK), were examined with a scanning electron microscope (JEOL JSM-6010 LA, JEOL, Tokyo, Japan) operating in a high vacuum mode. An accelerating voltage of 10kV was applied to capture SEM images.

## 3. Results and Discussion

Three grades of high density polyethylene selected for this study were linear grades varying in molecular mass and its distribution and in the level of branching. H3 shows a relatively low average molecular mass with a rather narrow distribution (M_w_ = 120,000 and M_w_/M_n_ = 2.2, respectively), while exhibiting slight branching—number of short chain branches (SCB) is below 5 per 1000 C atoms in the main chain. As found in previous studies [16], such a moderate level of branching suppresses crystallization, thus slightly reduces crystallinity compared to linear grades, but helps to maintain efficient stress transfer between lamellae, even thick extended-chain crystals, during mechanical tests. This facilitates ductile behavior of the material. While the previous experiments with more linear or lower molecular mass polyethylenes led also to extended-chain crystal morphology with the desired thickness range, the samples with thick crystals were reported to be brittle, fracturing at low strains [8,16]. The second grade—H4—is a highly linear HDPE, with molecular mass higher than H3, and polydispersity typical for HDPE’s (M_w_/M_n_ = 5.9). This very linear PE was expected to crystallize easier than H3, which should result in higher crystallinity, and formation of thicker and less defected lamellar crystals under pressure, yet possibly more prone to premature fracture due to lower concentration of inter-crystalline links, similarly to samples of lower molecular mass tested earlier [8,16]. The H5 of relatively high average molecular mass (M_w_ = 478,000), a broad weight distribution (M_w_/M_n_ = 12.2) and exhibiting rather low branching (below 3 branches per 1000 C) was also expected to develop crystallinity higher than H3 due to lower branching and very broad mass distribution, including the presence of low molecular fraction [35,36,37,38,39], form thick crystals upon crystallization at elevated pressure and perhaps demonstrate a ductile behavior (a sufficient amount of inter-crystalline links was expected to form due to short branches [36], as in H3). 

### 3.1. Morphology of Non-Deformed Samples

**Figure 2:**
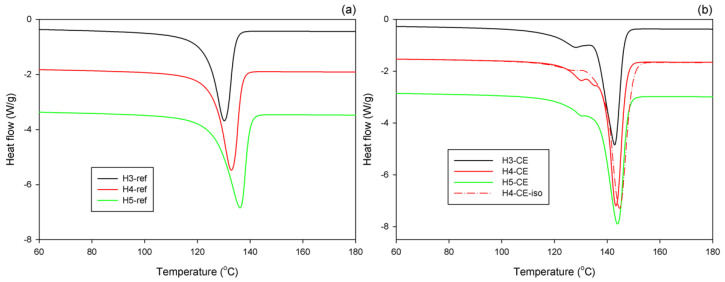
DSC melting thermograms of the reference samples, crystallized at conventional conditions (**a**), and CEPE samples, crystallized under pressure of 480MPa (**b**).

Figure 2 shows the DSC melting thermograms collected for the series of the reference samples, which were crystallized at conventional conditions (non-isothermal crystallization during fast cooling of the melt under near-atmospheric pressure), and CEPE-samples, crystallized non-isothermally under elevated pressure (cooling rate of 4 °C/min, p = 480 MPa). The results of evaluation of these curves are presented in Table 2.

It can be seen in Figure 2a that the reference samples of all three polymers exhibit a single melting peak, typical for high density polyethylene, with the peak temperature above 130 °C. The H3-ref sample shows the lowest melting peak temperature and crystallinity, probably due to the highest content of short branches in the chain (about five branches per 1000 C atoms in the chain, cf. Table 1), hindering slightly crystallization process. More linear chains in H4-ref and H5-ref facilitate formation of thicker crystals (higher T_m_) and higher overall crystallinity. The highest melting temperature (the thickest crystals) and the highest crystallinity were observed in H5-ref, which exhibits a high average molecular weight M_w_, yet a very broad molecular weight distribution (M_w_ = 478,000, M_w_/M_n_ = 12.2, respectively). This feature enables formation of relatively thick lamellae and reaching a high degree of crystallinity. 

Figure 2b shows that the CEPE samples, crystallized non-isothermally at high pressure, demonstrate the main melting peak with the maximum at T_m2_ = 142–144 °C, indicating the melting of very thick crystals, about 100–200 nm in thickness—cf. Table 2. Since the crystalline stem length in these crystals is much bigger than in conventional folded-chain crystals and approaches the average chain contour length (260–350 nm, as estimated from M_n_) such thick crystals are usually referred to as “extended-chain crystals” [28]. The crystals of pseudo-hexagonal phase were formed under high pressure instead of ordinary orthorhombic phase [27]. The high mobility of pseudo-hexagonal phase enabled a substantial crystal thickening and the formation of very thick, “extended-chain” lamellae. The crystals of pseudo-hexagonal phase were transformed to orthorhombic modification upon cooling, without reduction in size.

In addition to the main melting peak, a much smaller peak was observed on its low-temperature shoulder, at T_m1_ = 129–130 °C. This peak indicates the melting of much thinner crystals, similar to the ordinary folded-chain crystals formed at conventional crystallization conditions, as e.g., in the reference samples. These folded-chain crystals formed probably in the later stage of the high-pressure process, when the temperature decreased, well after formation of extended-chain crystals [26]. In fact, when the H4 was crystallized isothermally at T = 244 °C for a long time (sample H4-CE-iso) the size and the maximum temperature of the main melting peak of CE crystals (which formed first) increased, while the size of that secondary peak related to folded-chain crystals (grown later, upon cooling) decreased substantially, simply because much less material remained untransformed up to this stage; see Figure 2b.

Due to high mobility in pseudo-hexagonal phase leading to crystal thickening, a considerable fraction of pre-existing chain entanglements can be resolved during crystallization and crystal thickening. This leads to a significant increase of crystallinity [8,29], while the remaining part of the material, incapable of crystallization and constituting an amorphous component, may demonstrate an entanglement density either lower (many entanglements resolved—as perhaps in the case of linear chains of relatively low molecular weight) or higher than in the initial melt, as some entanglements might be redistributed (swept) from the growing crystal into the amorphous phase instead of being fully resolved [6]. This is confirmed by very high crystallinity of the CEPE samples—above 86 wt.%; in the case of slow, isothermal crystallization reaching even nearly 100 wt.% (H4-CE-iso). The small fraction of the folded-chain crystals grown in the final stage of the crystallization process (13–16 wt.%, as estimated for non-isothermally crystalized samples and only ca. 3 wt.% in H4-CE-iso; cf. Table 2) is probably formed in these regions of the melt where entanglements were still preserved or even concentrated, so the crystallization in the extended-chain fashion there was difficult. Ultimately, only a relatively small fraction of the polymer was unable to convert to the crystalline phase (either thick extended-chain or thin folded-chain crystals) due to the high concentration of entanglements or due to other constraints, and then formed an amorphous component. 

The highest melting point (presumably the thickest crystals) and the highest crystallinity were found in the samples of H4 (H4-CE and H4-CE-iso), which is essentially a highly linear PE. Samples H3-CE and H5-CE demonstrated a slightly lower melting temperature as well as crystallinity, probably due to higher branching, which made the crystallization process more difficult.

**Figure 3:**
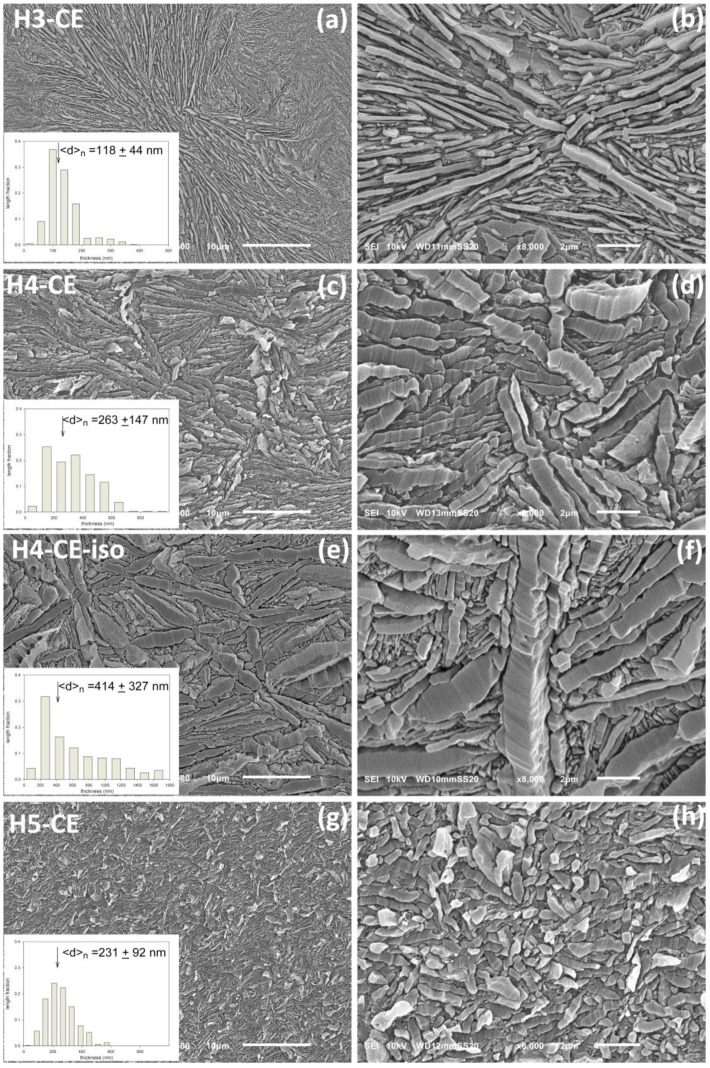
SEM micrographs of non-deformed CEPE samples etched with permanganic etchant: (**a**,**b**)—H3-CE, (**c**,**d**)—H4-CE, (**e**,**f**)—H4-CE-iso, (**g**,**h**)—H5-CE. Micrographs (**a**,**c**,**e**,**g**) were taken at magnification of 2500, while (**b**,**d**,**f**,**h**) show the respective samples at higher magnification of 8000. Insets show the lamella thickness distribution evaluated from the SEM micrographs.

The morphology of CEPE-samples was examined with scanning electron microscopy (SEM). Figure 3 presents the SEM micrographs of the non-deformed samples, etched with permanganic etchant in order to expose their lamellar morphology [34]. This treatment etches preferentially the amorphous material between lamellae, while the crystalline lamellae remain nearly undamaged, being etched a little deeper only locally, in points of high concentration of defects, e.g., dislocations [40]. The plane of observation was exposed for etching and subsequent observation, using the microtoming method, therefore there were no features in the micrographs associated with deformation, fracture or related damage to the material that might occur in samples prepared by other methods, e.g., freeze-fracturing. Therefore, the micrographs show probably the actual morphology of non-deformed samples. 

SEM observations demonstrated that the lamellae in the examined samples generally did not show any preferred orientation. Nevertheless, in order to better visualize the structure as well as for easier determination of lamella thickness, the micrographs chosen for presentation in Figure 3 were taken in selected areas where majority of the lamellae were oriented edge-on. Note an important feature: some parallel striations can be recognized on the exposed faces of the edge-on oriented lamellae, roughly perpendicular to the lamella plane; see e.g., Figure 3d,f. These striations, produced by slightly uneven etching of the crystalline phase (etching rate depends on the direction in the crystal and the perfection of the crystal), visualize the direction of the chain in the lamellae [40]. 

It can be seen from the presented micrographs that the lamellae in H3-CE and H4-CE are very thick, long—often much longer than 10 μm—and flat, with no twists. Usually, several adjacent lamellae are arranged parallel to each other and form stacks. These stacks are organized in higher-level radial structures resembling spherulites, radiating from the center of the spherulite and often extending up to the spherulite boundary. Frequently, especially in H3-CE, between two very thick lamellae one or more parallel but much thinner lamellae can be observed. The long, edge-on oriented lamellae in many instances give the impression to be shattered into shorter blocks. However, observations of tilted and flat-on oriented lamellae demonstrated that the apparent discontinuities seen at the lamellae edges are actually the result of locally deeper etching in places with high concentration of defects, such as e.g., dislocations [40]. This can create quite deep notches on the edge of an otherwise long and continuous lamella, which may then look likely to be broken into shorter pieces when observed in an edge-on orientation. 

In contrast, lamellae seen in H5-CE, are much shorter than in H3-CE or H4-CE—only about 2–5 μm in length—and while still arranged in parallel stacks, do not show clear higher level organization. Such crystallization habit is probably related to a significantly higher molecular weight of this PE grade comparing to H3 and H4. 

In all samples studied the lamellae were found extremely thick—the average lamella thickness, determined from micrographs (at least 400 lamellae measured in each image) ranged from about 120 nm in H3-CE to approx. 230 nm in H5-CE and above 260 nm in H4-CE—cf. Figure 3 and Table 2. Comparing these thicknesses to the contour length of the average chain, which is approx. 320, 260 and 350 nm for H3, H4 and H5, respectively (as estimated on the basis of M_n_), one can conclude that in the studied samples crystallized under high pressure, the chains were actually built into the crystals in a well extended fashion, with probably only occasional folding. Moreover, the lamellae observed in the sample H4-CE-iso, crystallized, and annealed isothermally for 1 h at high pressure, were found even thicker—up to 2 μm—with the average thickness of about 420 nm, which exceeds well the average chain length ≈ 260 nm. This indicates that the individual crystalline stems had to be frequently formed from more than one chain, and consequently some chain ends had to be incorporated inside the crystal. 

The average lamella thickness determined from SEM micrographs is noticeably larger than the crystalline stem length l* that determines the crystal thickness, estimated from the melting temperature with the Gibbs-Thomson equation (Equation (4))—see Table 2. This discrepancy demonstrates that a great caution is needed when using this equation for estimation of the thickness of lamellar crystals. It seems that the melting point data can only be used for a rough approximation of the crystal thickness, especially in the range of high melting temperatures, close to the equilibrium melting temperature, where the dependence of l* on T_m_ becomes very steep, and any single, even small, experimental error in T_m_ may result in a large inaccuracy in the estimation of lamella thickness. 

### 3.2. Deformation Behavior

The reference samples, crystallized under conventional conditions, as well as samples crystallized under high pressure, composed of extended-chain lamellae, were deformed at room temperature in the uniaxial compression mode with the constant true strain rate. All reference samples were found ductile as they deformed easily to high strains, well above e = 1.6. The H3-CE and H5-CE samples also appeared ductile in this range. In contrast, samples of H4-CE exhibited different behavior—while a few samples deformed in a ductile manner even to high strains, many others, especially those of slightly higher crystallinity, demonstrated brittle behavior, fracturing at low strains, usually prior to reaching the yield point. Moreover, practically all of the H4-CE-iso samples of extremely high crystallinity, appeared brittle. This brittleness was probably due to a strong deficit of amorphous chains in these samples, which could provide a sufficient connectivity between crystals through amorphous layers and transmit stress between these crystals, like tie-molecules or similar.

**Figure 4:**
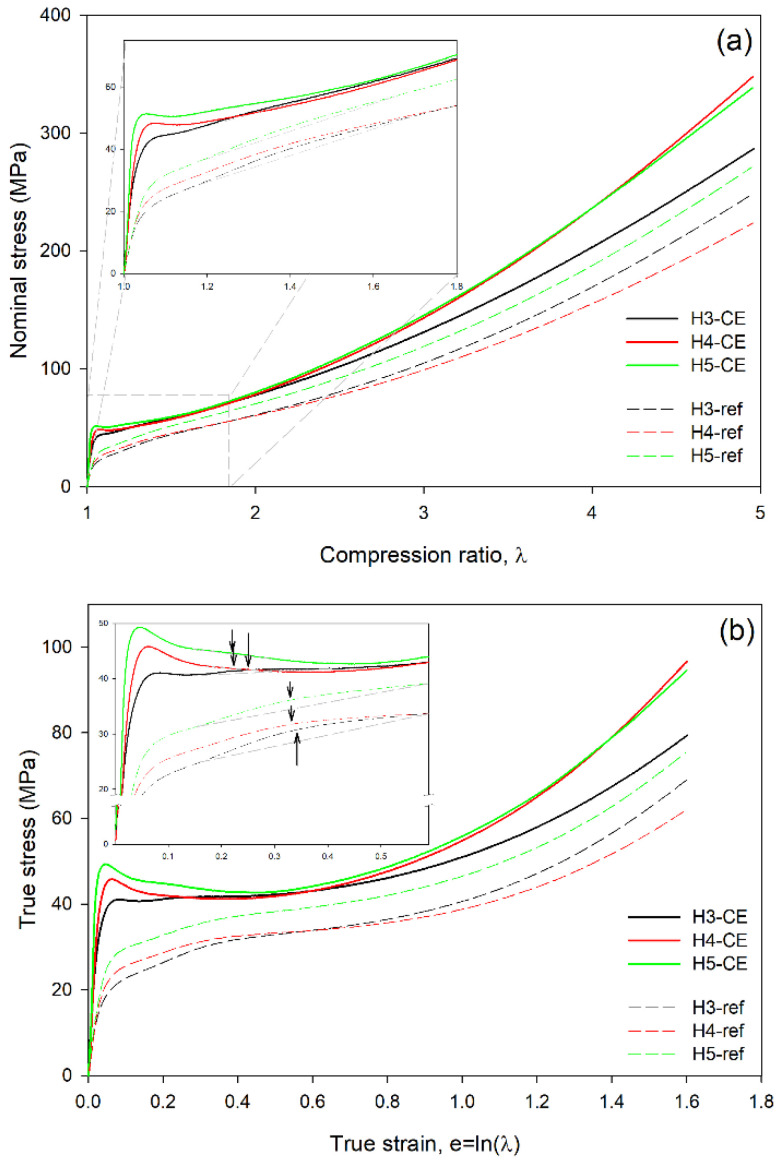
The experimentally determined dependencies of the nominal stress on the compression ratio (**a**) and the derived true stress-true strain curves (**b**). Equations (1) and (2) were used for calculations of true stress and true strain, respectively. Insets show enlarged initial part of the curves. Black arrows indicate the approximate location of the second yield.

Figure 4a presents the dependencies of the nominal stress vs. compression ratio determined experimentally in the reference and the CEPE samples. From these data the true stress-true strain curves were calculated using Equations (1) and (2) and plotted in Figure 4b. It can be seen that in the compression curves of the reference samples, crystallized at conventional conditions and containing thin folded-chain lamellae, both the nominal and true stress increase continuously with increasing deformation, without any maximum that could define the yield point (in such a case the yield can be alternatively estimated with the offset construction [41]). This is a typical response of polyethylene to compression [9]. In contrast, all CEPE samples show a maximum in stress defining the yield point, seen clearly especially in the true stress-true strain curves. The elastic modulus (determined from initial slope) and the stress at yield are notably higher in CEPE samples than in the respective reference samples, which reflects their higher crystallinity and thickness of crystal. The stress maximum is followed by a short strain softening region extending up to the true strain of about e = 0.5, where stress-hardening stage begins in all samples. Comparing the mechanical response of the reference samples, it can be seen that the stress in the yielding and plastic flow regions increase from H3-ref to H4-ref and then to H5-ref, which reflects the increasing thickness of the lamellar crystals in these samples (cf. Table 2). The crystal thickness is known to control the yielding and plastic flow behavior of semicrystalline polymers with relatively thin folded-chain crystals [16,42]. In the strain-hardening stage, e > 0.6, the stress-strain curves of all three control samples show similar slope, although H3-ref demonstrates slightly stronger hardening effect than H4-ref or H5-ref. Since the strain-hardening is controlled primarily by the density of the molecular network in the amorphous phase [6,11], the observed response of the reference samples can indicate similar properties of the molecular network, perhaps with a slightly higher network density in the H3-ref sample. On the other hand, the H3-CE sample with extended-chain crystals shows lower slope of the curve in this strain range, i.e., weaker strain-hardening, while H4-CE and H5-CE demonstrate similar strain-hardening, which appears stronger than in H3-CE or the reference samples. This may indicate that the density of the network of entangled chains in H3-CE is slightly lower than in the reference samples, whereas the concentration of entanglements in amorphous phase of H4-CE and H5-CE is a little higher, probably as a consequence of some redistribution of pre-existing entanglements into amorphous phase during formation and thickening of pseudo-hexagonal crystals under high pressure. High-pressure crystallization in H3, of molecular weight lower than H4 or H5, probably resulted in resolving of more chain entanglements, which led to a less dense molecular network in H3-CE sample than in the reference material or species of higher molecular weight, H4 and H5. 

Another feature observed in the true stress—true strain curves of the reference as well as CEPE samples is the so-called ‘second yield,’ which can be distinguished as a hump or bulge (low and very broad local maximum) extending from about e ≈ 0.2 to e ≈ 0.5 (centered around e ≈ 0.35) in the reference samples and located at slightly lower strains, from e ≈ 0.15 to e ≈ 0.35 (centered about e ≈ 0.22–0.25) in the CEPE samples; see the inset of Figure 4b displaying enlarged initial part of the stress-strain curves. 

The second yield, similarly to the primary yield, is generally associated with the deformation of the crystalline phase. Initially, the second yield was attributed to the activation of the block slip along the direction of the chain (so-called ‘coarse’ chain slip), which can ultimately lead to lamellar fragmentation [43,44,45,46,47]. Later, Sedighiamiri et al. [48] proposed to associate the second yield with activation of the transverse slip systems rather than with the coarse chain slip. In the recent studies [20,21,22] we demonstrated that the second yield is actually related to the microbuckling instability occurring in the stacks of lamellae that were initially oriented along the direction of compression. The lamellae of such specific, longnitudinal orientation are not able to deform according to the usual crystallographic mechanisms because the corresponding resolved shear stresses are much lower than critical. Consequently, the deformation is locally locked and the stresses increase with increasing strain. Then, the stacked lamella can respond to increasing compressive in-plane stress with bending or undulation, a phenomenon referred to as microbuckling instability [18,20]. Due to phase connectivity the neighboring lamellae are forced to bend jointly, which leads soon to development of bigger joint folds or angular kinks from a small initial ripple. Kinking, triggered by microbuckling, was observed in many semicrystalline polymers, e.g., polyethylene (PE), isotactic polypropylene (iPP), polyoxymethylene (POM) or isotactic polystyrene (iPS) [17,49,50,51,52,53]. 

The microbuckling in semicrystalline polymers is generally analogous to buckling in other layered systems. The layer buckling phenomenon was first investigated in structural geology. Several theories of rock folding were developed, mainly using the elastic approach (see, e.g., [54]). Later, similar considerations were made for liquid crystals and highly oriented block copolymers with lamellar morphology [55,56,57,58]. It was concluded that a necessary condition for buckling to occur in a layered structure consisting of alternating hard and soft layers is a large difference in the layer stiffness. In addition, a strong coupling between layers is also required if buckling is to occur in tension, as in the case of some semicrystalline polymeric materials. Buckling is driven by the simple, natural tendency of a material to deform in a way that minimizes energy—buckling of hard and soft layers proves less expensive in terms of free energy than the alternative mechanism by dilatation of soft layers or voiding at interface [59]. Buckling has been found to be controlled by strain. Read et al. [55] demonstrated that buckling starts at a critical strain due to a geometrical instability that can even happen in the elastic range. Such a small sinusoidal instability (undulation) continues to evolve into a fold, angular kink or a chevron shape as the strain increases above the instability point. Non-linear phenomena, such as the plastic deformation in hard layers, can be also involved in buckling and subsequent formation of the kink, thus enhancing its angular profile [55]. The development of sharp angular kinks or chevrons turned out to be energetically advantageous compared to round folds, especially if the fraction of the hard phase was high [59]. The elastic theories predict the dependence of the critical strain for buckling initiation on the ratio of the layer stiffness (determined by the product of layer thickness and its elastic modulus) of the soft and hard layers [18,55]. FEM model calculations performed for the oriented block copolymer structure [55] demonstrated that the first sinusoidal undulations were generated when the ratio of the elastic moduli exceeded 500 (at constant layer thickness). In semicrystalline polymers deformed above T_g_, this ratio is expected to be much higher (e.g., in polyethylene, PE, an overall modulus of elasticity of crystalline phase is about 50 GPa—elastic constants calculated for anisotropic PE crystal range from c_11_ ≈ c_22_ ≈ 10 GPa to c_33_ ≈ 300 GPa [60], while that of amorphous layer is below 40 MPa [61], i.e., smaller by 3 order of magnitude), which can indicate their high susceptibility to microbuckling upon deformation. 

Microbuckling is limited to the specifically oriented lamellae and can accommodate only a minor strain. Nevertheless, it appears to be a significant transformation in the deformation sequence, since kinking triggered by microbuckling results in a rapid and fairly large irreversible rotation of the lamellae that were involved in the kink. Such rotation leads to an increase of the resolved shear stress in the potential slip planes of the just reoriented lamellae, enabling activation of new deformation mechanisms of lower plastic resistance, such as crystallographic slip. It opens a new path of relatively easy deformation that allows a significant part of the material volume, previously locked, to join the deformation by relatively easy crystallographic mechanisms, similar to those previously available only for other lamellae already preferably oriented to slip, as e.g., those in diagonal parts of the spherulites. It was reported that microbuckling and kinking can also contribute to some fragmentation of lamellae, although limited only to kink tips [22]. 

Since microbuckling instability can effectively modify the deformation path, it often manifests macroscopically in the stress–strain curve as a characteristic low and broad hump, commonly referred to as the second yield. We reported in our previous papers [20,21,22,25] a hump related to the second yield in the stress-strain curves of several grades of polyethylene, observed around e ≈ 0.3–0.4 in the plane-strain compression. Concurrently, characteristic changes were observed in the 2D-SAXS and 2D-WAXS images, including the formation of a distinctive four-point SAXS pattern, which clearly indicated lamella kinking and development of a chevron-like morphology. 

Experimental evidence has shown that microbuckling is driven by significantly different levels of stiffness of the hard (crystalline) and soft (amorphous) layers and their strong interfacial connectivity [12,17,18,55]. It was found that the critical strain for microbuckling (i.e., the strain at which microbuckling is initiated) depends primarily on the ratio of stiffness of the layers, as well as on the concentration of the stress transmitters at the crystal–amorphous interface (like tie-molecules, entangled loops, etc.) [20,21]. The dependence of the critical strain of microbuckling on layer stiffness was confirmed experimentally for semicrystalline polymers—in the recent studies of deformation instabilities in samples of non-oriented and oriented PE [20,21,22,25], we found that microbuckling instability occurred in PE samples of various structure at the true strain ranging approximately from 0.3 to 0.4. The critical strain was found proportional to the ratio of the stiffness of the amorphous and crystalline layers, which is consistent with the general prediction. The stiffness of the crystalline and amorphous layers is determined by the thickness and the modulus of elasticity of the respective phase. In particular, when the elastic properties of crystalline and amorphous components have not been altered, the critical strain of microbuckling depends on the ratio of the thickness of the amorphous and crystalline layers, although the actual thickness of the layers can vary [20]. 

The hump assigned to the second yield in the reference samples, uniaxially compressed in this study, was observed at the position similar to that found previously in various PE samples deformed in the plane-strain compression [20,21,22,25]—centered around e ≈ 0.35. In contrast, a less distinct bulge, but centered at lower strains (e = 0.22–0.25), was discerned in CEPE-samples. The shift of the second yield to lower strains in CEPE seems reasonable, if we recall that the microbuckling is controlled by the ratio of stiffness of the amorphous and crystalline layers. The elastic moduli of the crystalline phase in the reference and CEPE-samples are identical, while the moduli of the amorphous phase are most probably very similar (as suggested by similar strain hardening behavior). Therefore, the key parameter for microbuckling should be the ratio of the layer thickness. As the crystallinity of the CEPE-samples is significantly higher than the reference samples, the thickness ratio *l_a_/l_c_* is clearly lower in the CEPE- than in the reference samples. This implies an earlier microbuckling in CEPE-samples that should occur at a lower critical strain, and perhaps the size of the hump associated with the second yield should be smaller than in the reference samples of conventional morphology.

### 3.3. Microscopic Observations

**Figure 5:**
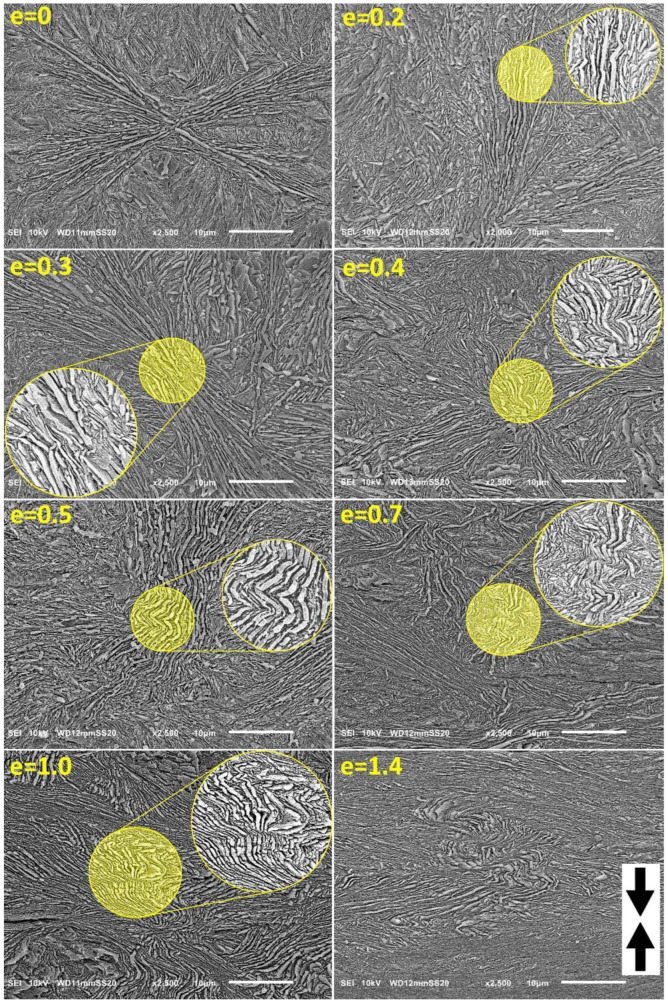
SEM micrographs of the H3-CE samples deformed by uniaxial compression to the true strain indicated. The direction of compression is vertical. The observation plane was exposed by microtoming followed by permanganic etching. Circular insets show enlarged details.

**Figure 6:**
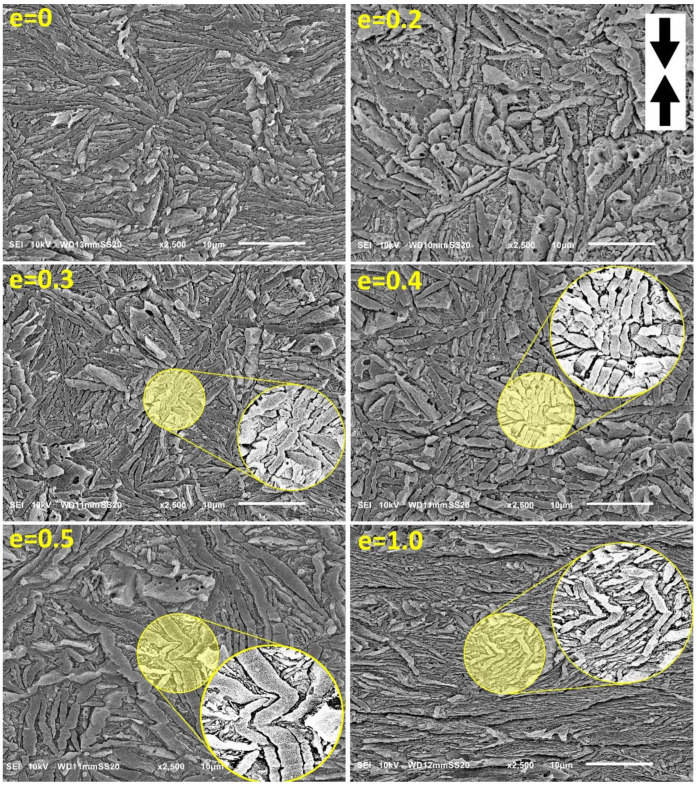
SEM micrographs of the H4-CE samples deformed by uniaxial compression to the true strain indicated. The direction of compression is vertical. Circular insets show enlarged details.

**Figure 7:**
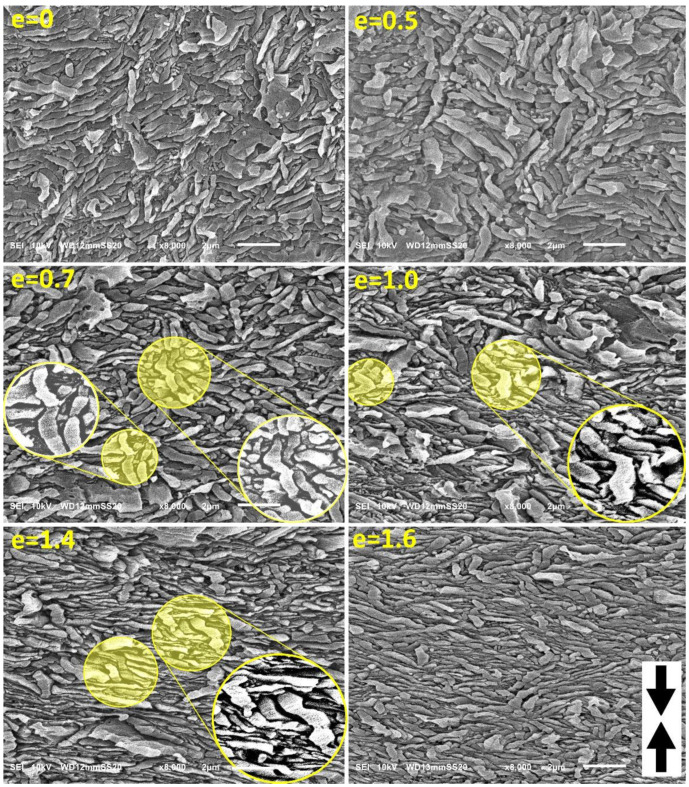
SEM micrographs of the H5-CE samples deformed by uniaxial compression to the true strain indicated. The direction of compression is vertical. Circular insets show enlarged details. Note, that the magnification is higher than that of the micrographs in Figure 5 and Figure 6.

Microscopic observation of the deformed samples was needed to characterize the structural changes accompanying deformation and obtain direct evidence of active deformation mechanisms, including microbuckling instability in CEPE samples during their deformation. For this purpose, microscopic observations of samples deformed to various strains were carried out using a scanning electron microscope. Figure 5 presents a set of SEM micrographs of the sample H3-CE, both non-deformed and deformed to strains ranging from e = 0.2 to e = 1.4. Figure 6 and Figure 7 present similar sets of SEM micrographs of deformed samples H4-CE and H5-CE. In all micrographs the loading (compression) direction is roughly vertical. The presented micrographs document well the evolution of lamellar morphology with increasing strain, including the formation of angular kinks in the lamellae that were initially oriented along the LD and the development of these kinks with increasing strain, which occurred in all PE grades studied here. Kinking appeared collective, involving several neighboring lamellae. The most clear picture of microbuckling, followed by the formation of collective lamellar kinks, can be seen on the micrographs of the H3-CE sample; cf. Figure 5. The lamellae in this sample prior to deformation were quite uniform in thickness, long compared to their thickness, straight and usually organized in long stacks of nearly parallel lamellae, which in turn, formed spherulites at a higher morphological level. The first signs of activity of the microbuckling can be recognized already at the strain as low as e = 0.2, where local undulations can be observed to arise, initially along individual lamella. Apparently, such instability spreads soon over the adjacent lamellae, which leads to a collective kinking of several neighboring lamellae (e > 0.3). It can be seen in the micrographs that the formation of angular kinks is preferred over round folds. This behavior is in line with the theoretical prediction that angular kinks, involving severe plastic deformation, should be energetically advantageous compared to round folds, especially when the fraction of the hard (crystalline) phase is high [59]. The development of such angular kinks, engaging several neighboring lamellae, can be observed at e = 0.3–0.4. A further increase in strain leads to a further development of kinks, tightening the apex angle at the kink tip and progressive rotation of lamellae in the kink limbs out of the loading direction. Frequently, several kinks develop along the length of the stacked lamellae, which give rise to a chevron-like morphology that gradually replaces the initial spherulitic morphology, at e ≥ 0.7. On the other hand, the other lamellae, oriented diagonally in spherulites, supposed to deform preferably by slip mechanism from the beginning (after passing the yield point), do not undergo any kinking and therefore remain straight up to high strains, only decreasing its thickness. This reduction in thickness and concurrent rotation of the chain direction inside lamella can confirm deformation by crystallographic slip along the direction of the chain as an active mechanism operating in these lamellae [1,2,3]. The morphology of the deformed material at higher strains consists then of relatively long straight lamellae and much shorter, but also straight, parts of the lamellae in the kink limbs, separated by kink tips. No damage to the lamellae in the tip regions was found, and the lamellae, although heavily deformed, mostly retained their integrity. Importantly, all of the straight sections of lamellae in kink limbs are oriented at an angle to the loading direction, which orientation favors chain slip, which becomes the primary deformation mechanism of nearly all lamellae beyond the microbuckling instability. The deformation instability, developing into kinks, was observed by SEM to occur in H3-CE at the true strain of e ≈ 0.2–0.3. This range of strains coincides well with the local maximum recognized in the stress-strain curve, centered around e ≈ 0.25 and assigned to the second macroscopic yield. It confirms again the hypothesis that the second yield is directly related to the microbuckling instability [20,22]. This instability occurs at the strain lower than that found previously in conventionally crystallized PE (e ≈ 0.35, as observed in the reference sample H3-ref or in the PE samples of various structure examined in the previous studies [20,21,22,25]). Such a reduction in the critical strain is associated with a substantial increase in the thickness of the crystalline layer relative to the amorphous layer and an increase in the crystallinity of the CEPE material, which results in a significant decrease in the ratio of the amorphous to crystalline layer stiffness, which has been shown to control primarily the microbuckling behavior. As a result, the critical strain of microbuckling instability decreases significantly, as expected [20,22]. 

As can be seen in Figure 6, microbuckling and kinking in the H4-CE sample is slightly less pronounced than in H3-CE (Figure 5). We did not find any indication of microbuckling at e = 0.2. However, at e = 0.3, some small lamellar kinks can be recognized, similarly to H3-CE. These kinks involve adjacent lamellae and develop, sharpening the apex angle, with increasing strain. The intensity of kinking and the average tilt of lamellae in kink limbs with respect to LD tend to be generally lower than in H3-CE at similar strain. These differences can be attributed to different initial morphology—lamellae in H4-CE are similar in length to these seen in H3-CE, but are much thicker, thus have a lower aspect ratio. This can appear to be another factor modifying microbuckling behavior—apparently, microbuckling is more intense when the aspect ratio of lamellae is higher. Less intense microbuckling and kinking resulted in a noticeably smaller hump in the stress-strain curve, assigned to the second yield in H4-CE (see Figure 4). On the other hand, this hump in H4-CE is located at strains slightly lower than that in H3-CE (e ≈ 0.22 vs. e ≈ 0.25, respectively). As the crystallinity in the H4-CE is significantly higher than in the H3-CE, the thickness ratio *l_a_/l_c_*, so the ratio of respective compliances, must be lower in H4-CE sample than in H3-CE. This implies a possible earlier initiation of microbuckling in the H4-CE. Unfortunately, this effect was not confirmed by direct SEM observations. However, in the case of less intense microbuckling, as in H4-CE, the events of its initiation could be not recognized or simply overlooked in sample deformed to only e = 0.2.

Figure 7 presents the set of SEM micrographs obtained for H5-CE sample at various deformation stages. The initial morphology of this material is different from H3 or H4 in that the lamellae are much shorter than in H3-CE or H4-CE, thus exhibit the lower aspect ratio, *L/l_c_* < 10. As a result, microbuckling and subsequent kinking, although present, is less intense than in the previously reported samples with much longer lamellae. The first kinks can be recognized in the micrographs not earlier than at the true strain of e = 0.5, while the mature kinks, resulting from cooperative deformation of adjacent lamellae, can be observed at e ≥ 0.7. Moreover, it seems that fewer neighboring lamellae are engaged in joint kinks. We attribute such a less intense microbuckling to the reduced aspect ratio of the lamellae in the structure, which perhaps results in weaker constraints imposed on the short lamellae in bulk. Consequently, a limited activity of other deformation mechanisms (as e.g., rotation of the lamellar stacks) may be allowed, especially for the shortest lamellae. Nevertheless, the long lamellae, oriented parallel to LD, still underwent microbuckling and then kinking similar to that in previously discussed samples. As in the case of H4-CE, a less intense kinking led to a less pronounced hump attributed to the second macroscopic yield in the stress-strain curve (see Figure 4).

**Figure 8:**
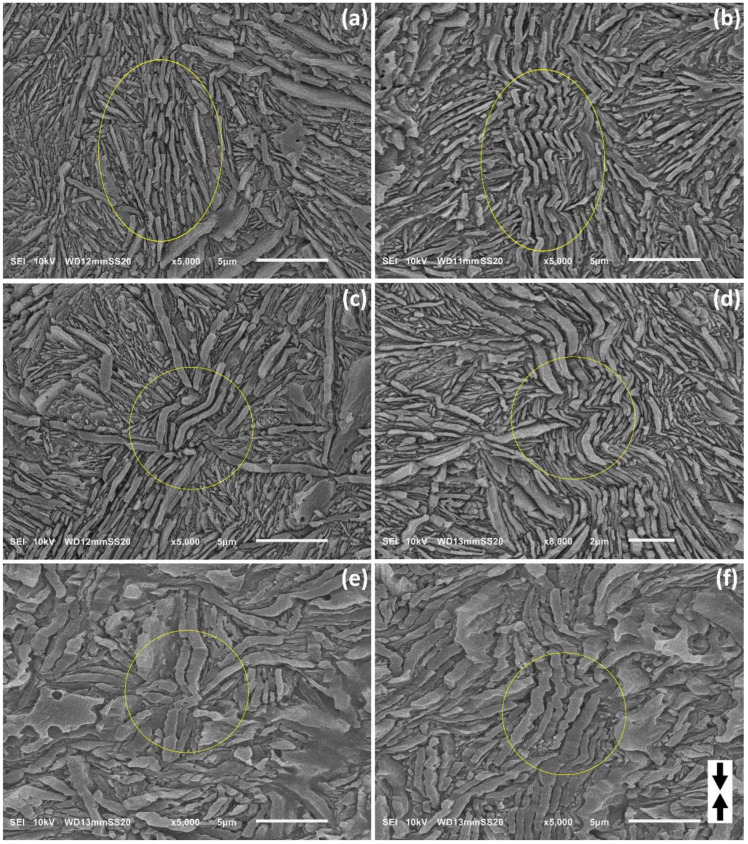
SEM micrographs of the samples H3-CE (**a**–**d**) and H4-CE (**e**,**f**) deformed by uniaxial compression, illustrating the microbuckling and early stages of kinking. The direction of compression is vertical in all micrographs. The true strain applied was: e = 0.2 (**a**), 0.4 (**b**), 0.4 (**c**), 0.7 (**d**), 0.5 (**e**), 0.5 (**f**). The observation plane was exposed by microtoming followed by permanganic etching. Areas of interest discussed in the text are marked with yellow circles.

The SEM micrographs of H3-CE and H4-CE samples deformed to relatively low strains were reviewed in order to find the mechanism of initiation of lamellar kinks. The selected micrographs are presented in Figure 8. Figure 8a—H3-CE at e = 0.2—evidences that the kinks are initiated and start to develop in long lamellae oriented along the direction of compression due to the instability leading to their undulation—small quasi-periodic ripples can be recognized along the individual lamellae already at e = 0.2; see Figure 8a. As the strain increases, the amplitude of the undulation increases and the instability is gradually transmitted to adjacent lamellae, so that several lamellae in a stack begin to deform cooperatively, creating a larger lamellar kink—see Figure 8b; e = 0.4. This mechanism is similar to that observed in buckling of several other materials [55,56]. 

Microbuckling instability can be additionally triggered in the lamellae under longitudinal loading by some fluctuations of their thickness — deformation resistance generally decreases with decreasing thickness of the lamella [42], therefore their locally thinner parts are more susceptible to any deformation instability and apparently microbuckling may be easier to initiate there. This effect can be observed in the sample H4-CE, demonstrating significant roughness of the lamella surface and varying thickness—see Figure 8e,f. It can be clearly seen in these micrographs that multiple small, but already sharp kinks were preferentially formed in thinner parts of the lamellae. Very sharp tips of these kinks indicate that localized plastic deformation by crystallographic slip mechanism had a large share in their formation. 

Another factor contributing to microbuckling instability and the formation of kinks is perhaps the non-uniformity of the stress field around the longitudinally compressed lamella. This stress field can be locally disturbed, for example, by the other lamella with very different orientation, which is in contact with the side of the lamella preferably oriented for buckling. The presence of such side lamella and perhaps its deformation or translation can then contribute to generation of an additional shear stress component normal to the plane of the considered lamella under compression. Such a local perturbation of the stress field may be large enough to trigger microbuckling instability in this part of the lamella. In fact, kinks were often observed to develop near the point where the stack of lamellae oriented initially along the loading direction was in lateral contact with another lamella of nearly perpendicular orientation, as can be seen in the micrographs of Figure 8c,d. In addition, it was also observed that stacks of inclined lamellae in the immediate vicinity of a longitudinally compressed lamellae, and undergoing extensive shear deformation, possibly locally exerted additional shear stress on these compressed lamella, which encouraged their microbuckling, and subsequent kinking. 

**Figure 9:**
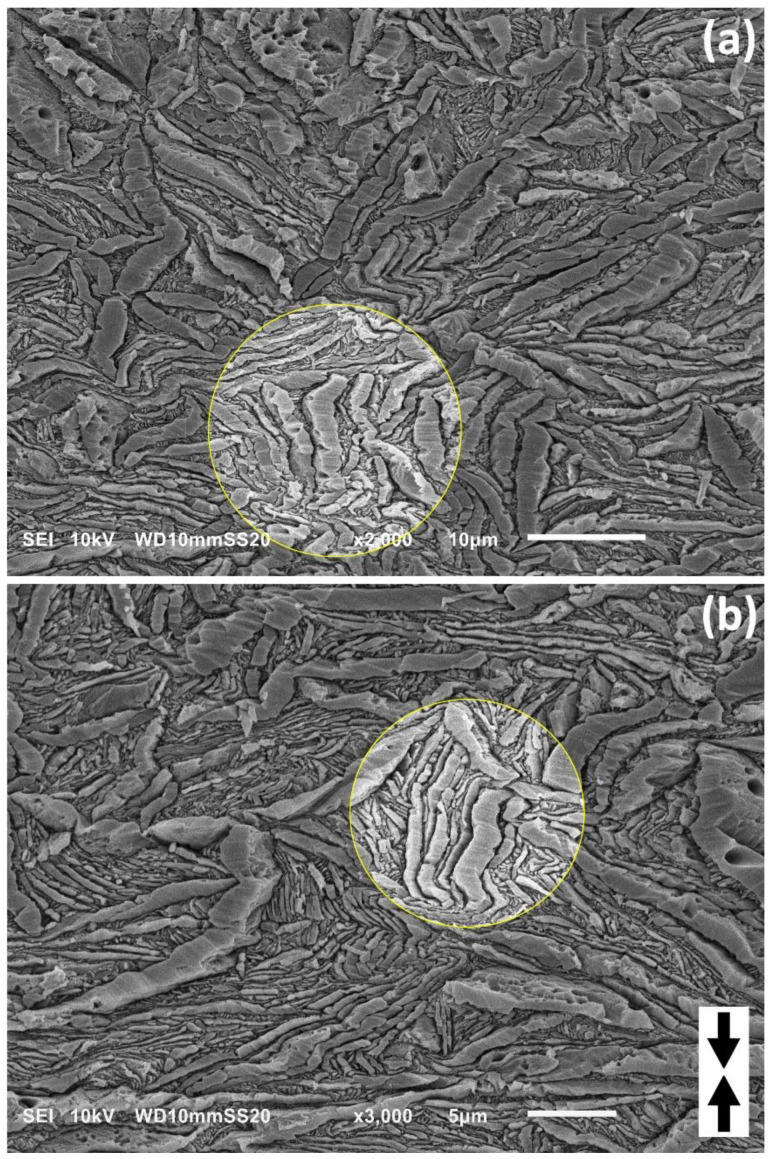
SEM micrographs of the H4-CE sample deformed by uniaxial compression to the true strain of e = 0.5. The micrographs (**a**,**b**) show two different regions of the same sample. The direction of compression is vertical. Sample etched prior to observation.

As mentioned above, the effect of plastic deformation due to crystallographic slip was noticed in the tips of the lamellar kinks. Further SEM observations of the deformed samples of H4-CE revealed that the same crystallographic slip system along the chain direction was involved throughout the entire kink, including both the tip region as well as the limbs. Figure 9 presents the SEM micrographs of H4-CE sample deformed to e = 0.5. In all thick lamellae oriented edge-on, parallel striations can be easily recognized—roughly perpendicular to the lamella plane in the initial non-deformed material (cf. Figure 3d–f and evidently tilted with respect to the lamella plane in the deformed samples (Figure 9). As already discussed, these striations reflect the actual direction of the chain in the lamella. The striations visible in practically all lamellae of the deformed sample—including these inclined that remain straight during the deformation as well as those which were initially oriented parallel to the loading direction and later underwent kinking—are all oriented preferentially in nearly the same direction, tilted towards the transverse direction. Such preferred tilted orientation of the chain direction (rotated inside individual lamellae out of the initial orientation) within the straight, diagonal lamellae and thinning of these lamellae with increasing tilt evidences the activity of the crystallographic chain slip as the primary deformation mechanism in these lamellae [1,3]. The same chain slip mechanism had to be, however active, also in the lamellae that developed kinks. The same uniform orientation of the chain direction throughout the entire kinked lamellae clearly indicates a well advanced single chain slip system. Its operation as a primary active deformation mechanism facilitated the development of sharp angular kink with straight limbs from an initial small undulation, which possibly may have started with some elastic bending. This crystallographic slip mechanism was not allowed at the very beginning of the deformation process because the resolved shear stress along the chain direction in the longitudinally loaded lamella was too low to activate this slip mechanism. However, once microbuckling produced the first undulations, the corresponding resolved shear along the chain direction started to increase locally due to modification of the lamella shape, resulting in some rotation of the chain direction. This, at some point, enabled the chain slip mechanism to be activated. Subsequently, this relatively easy deformation mechanism became to dominate further plastic deformation, resulting in development of mature angular kinks with straight legs. 

**Figure 10:**
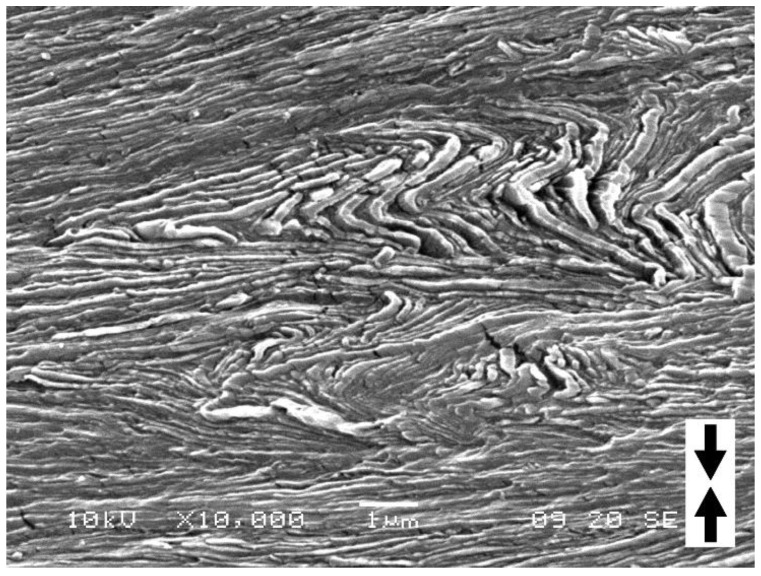
SEM micrograph of the H3-CE sample deformed by uniaxial compression to the true strain of e = 1.5 showing details of the mature kink. The direction of compression is vertical. Sample was etched prior to observation.

Figure 10 illustrates the further development of the lamellar kink with increasing strain (here the applied true strain e = 1.5). It can be observed that in this well advanced angular kink seen in the micrograph, the lamellae are continuous, not disrupted or broken even at the sharp tip of the kink, where they change their orientation abruptly. The lamellae are significantly thinned in the straight limbs of the kink, which again confirms their intense deformation by chain slip mechanism. Due to compressive load and shearing of the amorphous layers that accompanied the slip within crystalline lamellae (referred to as interlamellar slip [1,3]), adjacent lamellae are very close each to the other in the straight limbs. The intense intra- and interlamellar shear within both limbs of the kink quite often forced the local delamination and the formation of very small cavities between lamellae in the apical region, where the shear strain was smaller than in the limbs. However, such a local separation of the lamellae at the kink tip allowed close packing of the entire lamellar structure to be maintained without fracturing the lamellae. In addition, it can be observed that the apex angle of successive lamellae changes gradually across the kink and the kink becomes sharper with distance from the initiation point due to the strong deformation of adjacent lamellae by intense chain slip. This results in bigger cavities created between lamellae in the apex region at the far edge of the kink than in its center. The cavitation of the material was limited to the apical line of kinks described here and was not observed elsewhere in the deformed material, so globally it can be considered a marginal effect. This behavior observed in compression is opposite to tensile deformation, where extensive cavitation often plays much more important role. 

**Figure 11:**
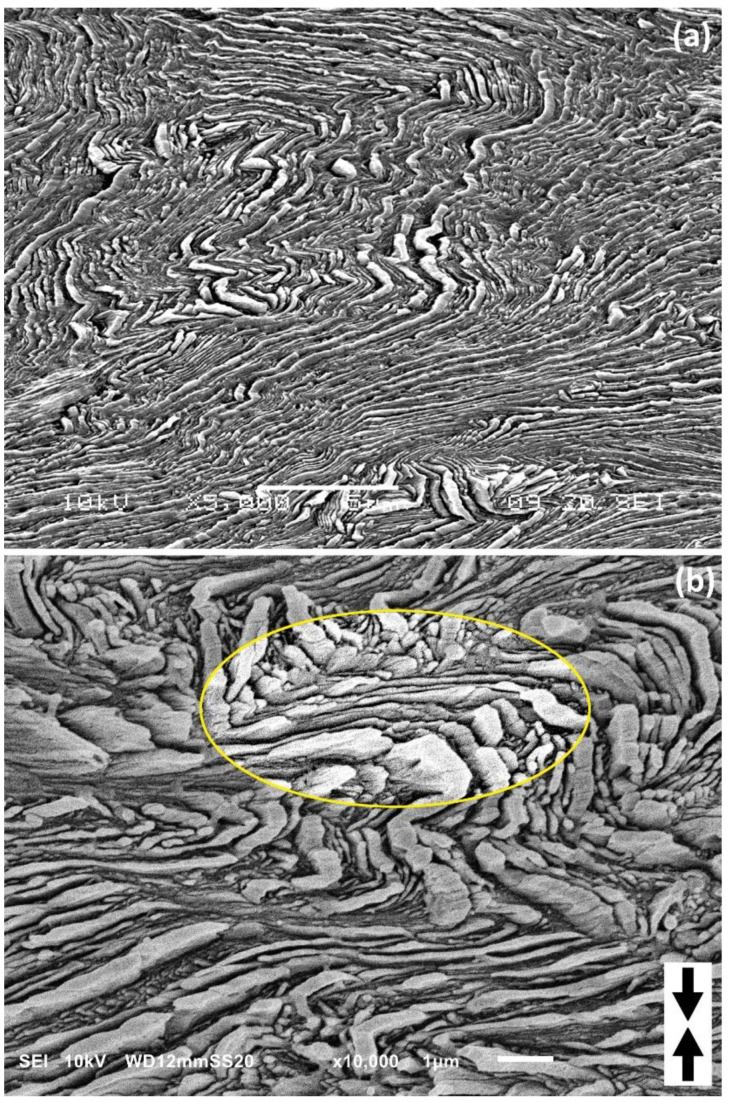
SEM micrographs of the H3-CE samples deformed by uniaxial compression to the true strain of e = 1.6 (**a**), e = 1.4 (**b**). The direction of compression is vertical. Samples were etched prior to observation.

Figure 11 presents another micrograph of a H3-CE sample deformed to high strains, e = 1.4–1.6. These micrographs show a very interesting feature of the deformation of samples with crystals of extended-chain morphology: the lamella preserved their continuity up to very high strains, e > 1.6 (compression ratio λ > 5). All lamellae—including these initially diagonal, which deformed from the beginning by chain slip mechanism supported by interlamellar slip, as well as those initially parallel to the LD, which first kinked due to microbuckling instability, and then continued to deform by chain slip—do not show any fragmentation and retain their integrity up to high strains; cf. Figure 11a. As can be seen in the inset of Figure 11b, the deformed lamellae were exceptionally thinned due to advancing slip, yet did not fragment even at very high strain. This behavior is very different from that of the conventionally crystallized samples consisting of relatively thin folded-chain lamellae, where a severe fragmentation of lamellae is usually observed during deformation in both tension and compression [6,20,62]. It was found that lamella fragmentation occurs in polyethylene in the true strain range of e = 0.6–1.0 (relatively weak at e = 0.6, but extensive at e = 1) as a result of slip instability, which results in its strong localization and finally leads to the lamella rupture [20]. The lamellae were already well thinned at this stage of the deformation process due to progressing chain slip. Any fluctuation in the lamella thickness or stress concentration at the crystal-amorphous interface caused by the highly stretched ‘stress transmitter’ chains ST in the adjacent amorphous layer, quickly led to the localization of the crystallographic slip in a narrow zone. Further intense slip, however limited to such narrow zones, led very soon to tearing of the already very thin lamella, as the total translation of the crystalline stem relative to the adjacent stem due to slip mechanism approached stem length. Consequently, an extensive fragmentation of lamellae was observed. In the case discussed here, the extended-chain lamellae are one order of magnitude thicker than a folded-chain crystals, therefore a much higher amount of shear is required do tear of such a very thick lamella, even if the slip localizes (actually, the lamella deformation in samples of extended-chain crystal morphology was found relatively homogeneous and only a very few traces of severe slip localization were noticed). As a result, the thick, extended-chain lamellae can be heavily deformed by intense slip and still maintain their integrity. Especially if the concentration of the ST chains, which ensure connectivity of crystalline and amorphous phases, but impose some constraints on extensive crystal deformation, is increased, as probably in the case of H4-CE and H5-CE. Greater concentration of ST chains results in a smoother distribution of the stress at interface, and thus reduced susceptibility to slip instability that would result in slip localization and eventual crystal fragmentation [20,21]. 

## 4. Conclusions

This work employed a set of polyethylene lamellar microstructures with extended-chain morphology. Samples containing the extended-chain crystals of the average thickness varying from 118 to 414 nm were obtained by crystallization at high pressure of 480 MPa. They were deformed to various strains by uniaxial compression at room temperature. The accompanying structural changes were analyzed using scanning electron microscopy (SEM). 

Similarly, to the conventionally crystallized samples with chain-folded morphology of lamellar crystals, some deformation instabilities were observed also during the deformation of extended-chain lamellae. It was found that at the true strain of e = 0.2–0.3 lamellae oriented specifically along the loading direction underwent microbuckling instability, leading to their cooperative kinking and ultimately to a chevron-like morphology. Microbuckling instability and subsequent kinking have a great influence on further deformation, as well as the resulting structure—lamellar folds or angular kinks induced by microbuckling instability result in a rapid and irreversible reorientation of the involved lamellae, which opens up for them a new path of easy plastic deformation by crystallographic slip, similar to that already operating in other lamellae, as e.g., those oriented diagonally. This facilitates the sample to accommodate strain in an energy-minimizing way. Macroscopically, the microbuckling instability shows up as the ‘second yield’ in the form of a small hump in the true stress–true strain curve. Microbuckling occurs in samples with extended-chain lamellae notably earlier than in structures with folded-chain lamellae, studied previously (e = 0.3–0.4). It was established that for a given stiffness of layers the initiation of microbuckling depends on the ratio of the amorphous to crystalline layer thickness *l_a_/l_c_,* which is significantly lower in highly crystalline samples with very thick extended-chain crystals than in PE crystallized at conventional conditions.

Microscopic observations demonstrated that microbuckling instability begins with small undulation of long lamellae under longitudinal loading—small periodic ripples can be recognized along the individual lamellae already at e ≈ 0.2. The amplitude of the undulation increases with increasing strain and the instability is gradually transmitted to adjacent lamellae, so that several lamellae in a stack begin to deform jointly, creating a larger lamellar kink. The mechanism is similar to that observed in buckling of several other materials. It seems that microbuckling instability is easier to initiate when the lamellae are very long and flat. Moreover, the instability can be additionally triggered by some fluctuations in the thickness of lamella, since the deformation resistance tends to decrease with decreasing thickness. Another factor contributing to microbuckling instability is perhaps the non-uniformity of the stress field around the longitudinally compressed lamella. This stress field can be locally disturbed, for example, by the other lamella with very different orientation, touching the side of the lamella oriented for buckling. Its deformation or translation can result in a local modification of the stress field that may be large enough to prompt microbuckling instability. 

The development of the lamellar kink from the initial small undulation, which possibly may have started with some elastic bending, proceeds through intense plastic deformation. The same crystallographic slip system along the chain direction was found to operate throughout the entire kink, including the tip region as well as both limbs. This slip activity facilitates the development of sharp angular kink with straight limbs from an initial small bend. The lamellae in the limbs become significantly thinned due to chain slip. The intense slip supported by interlamellar shear in both limbs sometimes forced the opening of very small cavities between lamellae in the apical region, where the shear strain was smaller than in the limbs, yet the dilatational stress was high. However, such a local lamellae separation at the kink tip allowed to maintain close packing of the entire lamellar structure without rupturing the lamellae. The cavitation was limited to the apical line of kinks and was not observed elsewhere in the deformed material, so globally it can be considered a marginal effect. This behavior observed in compression is opposite to tensile deformation, where extensive cavitation often plays much more important role. 

In contrast to conventionally crystallized samples, where extensive fragmentation of thin folded-chain lamellae due to slip instability is observed, usually in the range of true strain range e = 0.6–1.0, thick extended-chain lamellae deform stably and do not undergo any fragmentation up to very high strains, e > 1.6 (compression ratio of λ > 5). All lamellae—including those initially diagonal, which deformed from the beginning by the chain slip mechanism supported by interlamellar slip, as well as those initially parallel to the LD, which first bend or kinked due to microbuckling instability, and then further deformed by chain slip—did not show any fragmentation and retain their integrity. The deformed lamellae, although very much thinned as a result of well advanced slip, did not break even at very high strain, which demonstrates the stability of the deformation of thick lamellae by chain slip. This stability is related to large thickness of extended-chain lamellae, which are one order of magnitude thicker than folded-chain crystals. As a result, a much higher amount of shear is required for possible strong localization of the slip and then the disruption of such a very thick lamella, so that the slip may proceed almost unimpeded up to high overall strain. 

## Figures and Tables

**Table 1:** Molecular characteristic of polyethylene samples used [9].

Sample Label	M_w_ (g/mol)	M_w_/M_n_	Number of Branches (1/1000 C)	MFR (190 °C, 2.16 kg) (g/10 min)	Density (g/cm^3^)
H3	120,000	3.4	<5	2.3	0.952
H4	170,000	5.9	<0.2	0.2	0.962
H5	478,000	12.2	<3	-	0.954

**Table 2:** Characteristics of the reference and CEPE samples of polyethylene derived from DSC and SEM results.

Sample Code	T_m1_ (°C)	T_m2_ (°C)	X_c_ ^(1)^ (wt.%)	X_c_ (CF) ^(2)^ (wt.%)	X_c_ (CE) ^(2)^ (wt.%)	Crystalline Stem Length, l* ^(3)^ (nm)	Number- Average Lamella Thickness, *<d>_n_* ^(4)^ (nm)
H3-ref	130.3		61.1	61.1	0	17	
H4-ref	132.9		65.2	65.2	0	21	
H5-ref	136.2		70.9	70.9	0	28	
							
H3-CE	128.8	142.8	86.0	16.2	69.8	97	118 ± 44
H4-CE	129.3	144.2	98.2	13.7	84.5	200	263 ± 147
H5-CE	130.6	143.9	90.3	13.5	76.8	164	231 ± 92
							
H4-CE-iso	129.3	144.7	99.4	3.0	96.4	325	414 ± 327

(1) Overall crystallinity, calculated from the heat of fusion (DSC), with Equation (3). (2) Crystallinity of the CF (thin, folded-chain crystals) and CE (thick, extended-chain crystals) fractions estimated by deconvolution of the melting peak (DSC). (3) Crystalline stem length, calculated from the temperature of the melting peak with Equation (4). (4) Number average lamella thickness, measured in SEM micrographs (each dataset n > 400).

## Data Availability

The data presented in this study are available on request from the corresponding author. The data are not publicly available due to being stored in an internal repository.
